# Radial fan-based CO_2_ insufflation during laparoscopic surgery: a first-in-human study

**DOI:** 10.1007/s00464-026-12568-0

**Published:** 2026-01-12

**Authors:** A. I. de Jong, E. Ghilotti, F. Sterke, W. van Weteringen, P. J. Tanis, B. P. L. Wijnhoven, R. M. H. Wijnen, R. L. Dellacà, J. Vlot

**Affiliations:** 1 Department of Pediatric Surgery, Erasmus MC Sophia Children’s Hospital, University Medical Center Rotterdam, Rotterdam, The Netherlands; 2 Department of Electronics, Information and Bioengineering, Politecnico di Milano University, Milan, Italy; 3 Department of Biomechanical Engineering, Delft University of Technology, Delft, The Netherlands; 4 Department of Anesthesiology, Erasmus MC, University Medical Center Rotterdam, Rotterdam, The Netherlands; 5 Department of Surgery, Erasmus MC, University Medical Center Rotterdam, Rotterdam, The Netherlands

**Keywords:** Laparoscopic surgery, Radial fan, Insufflation, Innovation, Pneumoperitoneum, Pressure stability

## Abstract

**Background:**

Intra-abdominal pressure during laparoscopic insufflation with pressurized carbon dioxide (CO_2_) gas is strongly influenced by mechanical ventilation. Resulting pressure fluctuations can destabilize the surgical workspace and potentially cause harm associated with high insufflation pressures. To address this, a novel CO_2_ insufflator was developed using a radial fan and a gas reservoir to generate and maintain continuously stable insufflation pressures (radial fan-based insufflator, RFBI). This first-in-human study evaluated its safety and feasibility during laparoscopic surgery.

**Methods:**

Adults undergoing elective intraperitoneal laparoscopic procedures were included. All procedures were performed using the RFBI and an 11 mm study trocar. Primary outcomes were safety, defined as the absence of serious or harmful adverse device effects (SADEs or ADEs), and feasibility, defined as completing the procedure without switching to a conventional insufflator. Secondary outcomes included pressure stability at the device outlet and documentation of observed events affecting pressure stability (e.g., trocar insertion/repositioning, leaks, suction, etc.).

**Results:**

Twelve patients were enrolled, having a total RFBI insufflation time of 35.9 h in seven different laparoscopic procedures. No SADEs occurred. One ADE occurred while inserting a 5 mm instrument into the 11 mm study trocar that resulted in high air leakage, causing temporary loss of surgical workspace but no harm. All procedures were completed without the need to switch to a conventional insufflator. Pressure remained stable at both target pressures of 10 mmHg (median 10.0 mmHg, interquartile range (IQR) 0.12) and 14 mmHg (median 14.0 mmHg, IQR 0.15). The RFBI rapidly re-established the target pressure after observed events affecting stability, without manual intervention or procedural delay.

**Conclusion:**

This first-in-human study demonstrates that RFBI technology is safe, feasible, and capable of maintaining stable insufflation pressures across varied adult laparoscopic procedures. Radial fan-based insufflation effectively compensated for pressure fluctuations from ventilation and surgical events, warranting further evaluation of clinical benefits.

Laparoscopic surgery requires the creation of a capnoperitoneum by insufflating pressurized carbon dioxide (CO_2_) gas into the abdominal cavity to create a surgical workspace. Although indispensable to minimally invasive procedures, this capnoperitoneum has physiological consequences on the cardiovascular and respiratory systems [1–4]. Elevated intra-abdominal pressure is transmitted upward into the thoracic cavity, where it can alter cardiac filling, impede venous return, and reduce lung compliance. Mechanical ventilation further compounds these effects, producing cyclical increases in intrathoracic pressure, which displaces the diaphragm downward into the abdominal cavity, transiently elevating intra-abdominal pressure. This competition between ventilation-generated and insufflation-generated pressures often necessitates higher peak ventilation pressures to deliver adequate ventilation tidal volumes [5, 6]. Importantly, the insufflation pressure fluctuations are not solely the result of this competition, but are amplified by the design limitations of most current insufflation systems.

First developed in the 1950s, conventional automated insufflators use valves to reduce CO_2_ gas pressure from a high-pressure source, such as a cylinder or wall-mounted inlet, to the target pressure set on the device. By controlling relief and supply valves, the insufflation pressure is maintained within a range relatively close to the target value. However, this control is generally too slow to react to fast pressure changes such as those imposed by diaphragmatic movement during mechanical ventilation or the manipulation of surgical instruments. As a result, fast perturbations can directly increase the intra-abdominal pressure above the target pressure. These transient pressure peaks can have physiological effects similar to those of sustained high insufflation pressures, which have been associated with postoperative pain, reduced organ perfusion, and inferior vena cava compression [7]. The negative effects of this insufflation technology, which essentially has remained unchanged for decades and continues to be the standard of care today, emphasize the need for insufflation systems capable of maintaining stable, physiologically safe pressures despite the challenge of constant disturbances introduced by ventilation and surgical manipulation during laparoscopic procedures. The introduction of insufflation systems with a valveless trocar has provided improved pressure stability over conventional insufflators [8, 9]. Unfortunately, these open systems have several potential drawbacks, including entrapment of room air during suctioning and the release of smoke particles into the operating room.

To improve intra-abdominal pressure stability under dynamic operative conditions, we developed a radial fan-based CO_2_ insufflator (RFBI). Unlike conventional valve-based systems that deliver gas intermittently, the RFBI generates and regulates insufflation pressure using a high-speed radial fan in combination with an internal low-pressure, high-compliance gas reservoir. This configuration enables the device to respond rapidly to pressure fluctuations by continuously modulating fan rotational speed. Control adjustments occur within milliseconds, allowing precise alignment of delivered pressure with the target setpoint, even when challenged with abrupt external pressure changes.

A key feature of the RFBI is its ability to facilitate bidirectional gas flow between the insufflated cavity and the integrated reservoir. This allows excess gas to flow back into the device when the intra-abdominal pressure rises, for example, during diaphragm expansion in the inspiration phase, and to return to the cavity when pressure drops, such as during diaphragm retraction in the expiration phase. Therefore, the RFBI aims to minimize these transient pressure peaks and troughs, maintaining a physiologically safe and stable capnoperitoneum throughout surgery.

This first-in-human study was conducted to evaluate the safety and feasibility of the RFBI during laparoscopic surgery in adult patients. The primary endpoints were the absence of serious or harmful adverse device effects and the ability to complete the surgery without reverting to a conventional insufflator. Secondary objectives were the evaluation of the radial fan-based insufflation technique by assessing insufflation pressure stability at the device outlet and registration of intraoperative events that affected pressure stability.

## Materials and methods

### Ethical and legal aspects

The protocol was approved by the Medical Ethical Research Committee of Erasmus MC, University Medical Center Rotterdam, Rotterdam, the Netherlands (MEC-2023–0807), and registered on ClinicalTrials.gov (NCT06319053). All participants provided written informed consent before study enrollment. The RFBI evaluated in this study was designed and manufactured in-house at Erasmus MC, University Medical Center Rotterdam, Rotterdam, the Netherlands, and was used under investigational medical device regulations.

### Study design and patient enrollment

This was a single-arm, first-in-human study. Adults scheduled for elective intraperitoneal laparoscopic surgery were eligible for inclusion if a primary 11 mm study trocar could be used. Exclusion criteria were pregnancy or direct conversion to open surgery. Once enrolled, the laparoscopic phases of the surgery were performed using the RFBI. Anesthetic and surgical approaches followed standard practice and were not altered by study participation. As the study focused on evaluating intraoperative safety, feasibility, and performance, no postoperative follow-up beyond routine care was performed.

### Primary and secondary endpoints

The primary outcomes were safety and feasibility of the RFBI during adult laparoscopic procedures. Safety was defined as the absence of serious adverse device effects (SADEs) and of adverse device effects (ADEs) resulting in harm to the patient or device user. An ADE was any undesirable event related to RFBI use that affected the patient or device user. A SADE, as defined by the Central Committee on Research Involving Human Subjects of the Netherlands, was any ADE that resulted in death, life-threatening harm, permanent impairment of a body structure or function, hospitalization or its prolongation, or required intervention to prevent such outcomes. Feasibility was defined as the ability to insufflate the abdominal cavity without the need to switch to a conventional insufflator because of insufficient performance or malfunction of the RFBI. The secondary outcomes included the evaluation of the radial fan-based insufflation technique by assessing insufflation pressure stability at the device outlet and registration of intraoperative events that affected pressure stability.

### Operating room setup

**Fig. 1:**
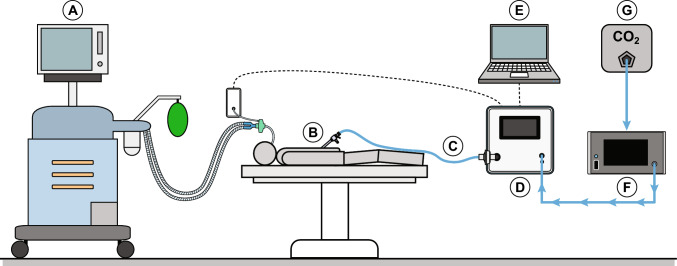
Setup in the operating room. A) Mechanical ventilator, B) trocar, C) insufflation tube, D) radial fan-based CO_2_ insufflator, E) researcher laptop, F) CO_2_ gas pressure reduction system, G) CO_2_ wall-outlet

The operating room setup was identical to standard laparoscopic practice, with the following additions for the study: the RFBI device and its accessories, a data cable connected to the mechanical ventilator, a custom-made pressure sensor connected to the patient’s airway opening to measure ventilation pressures, and a laptop running data acquisition software (Fig. 1).

### The radial fan-based CO_2_ insufflator

The RFBI generates and regulates insufflation pressure using a high-speed radial fan (U65MN-024KD-5, Micronel AG, Tagelswangen, Switzerland), enabling rapid, real-time adjustments in response to pressure changes in the abdominal cavity. Radial fan technology is established in the field of mechanical ventilation. In the RFBI, the radial fan allows bidirectional gas flow between the abdominal cavity and an integrated gas reservoir. This bidirectional flow capability enables precise and dynamic pressure control: When the intra-abdominal pressure is below the target pressure value, the radial fan increases speed to move gas into the abdominal cavity until the target pressure is reached. When the insufflation pressure exceeds the target pressure, the radial fan reduces speed, allowing excess gas to passively flow back from the abdominal cavity toward the device into the integrated gas reservoir (anesthesia bellow 1500–3378-000, GE HealthCare, Hoevelaken, the Netherlands).

**Fig. 2:**
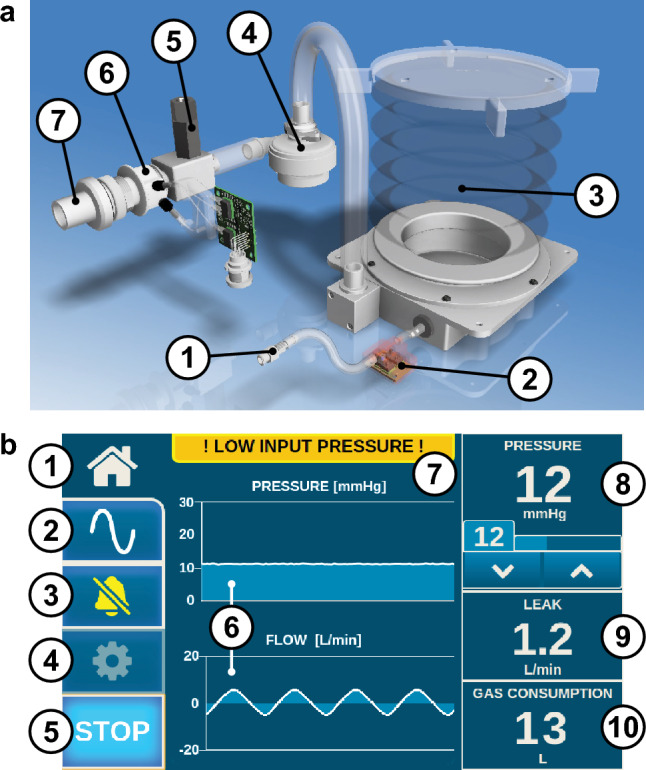
Gas path within radial fan-based insufflator and graphical user interface. **a** Gas path within the radial fan-based insufflator: 1) gas inlet; 2) reservoir pressure sensor; 3) low-pressure reservoir; 4) radial fan; 5) safety release valve; 6) pressure and flow sensor; 7) device outlet. **b** Graphical user interface main screen: 1) home button; 2) advanced menu; 3) mute alarms; 4) settings menu; 5) start/stop insufflation; 6) pressure and flow diagrams; 7) alarms display; 8) pressure measurement, target, and buttons; 9) leak flow; 10) CO_2_ consumption

Figure 2 shows the gas path within the RFBI. Gas enters the device at low pressure, fills the integrated reservoir, and is then pressurized by the radial fan before exiting through the outlet of the device. Insufflation pressure at the outlet is continuously measured by a differential pressure sensor (HTDM050BEQ, First Sensor AG, Berlin, Germany). This signal is processed by an embedded microcontroller (CY8C5888LTI-LP097, Cypress Semiconductor Corporation, San Jose, USA), which adjusts the radial fan’s speed via a servo controller (Escon Module 50/5, Maxon Motor AG, Sachseln, Switzerland) to maintain the target pressure. The control loop operates rapidly, ensuring stable pressure regulation. Device data and operational parameters are sent to the user interface, displayed on a built-in touchscreen (7-inch Raspberry Pi Touch Display controlled by a Raspberry Pi 4 Model B, Raspberry Pi Foundation, Cambridge, United Kingdom). This allows the surgical team to monitor key insufflation parameters and adjust settings directly from the display.

### Accessories

The following accessories were used with the RFBI to perform insufflation during laparoscopic surgery.

- **CO**_**2**_** supply system**: A conventional insufflator (Endoflator 40 / UI400, Karl Storz GmbH & CO. KG, Tuttlingen, Germany) was used in-line as a gas pressure reduction system (Fig. 1F) to reduce the institutional CO_2_ wall-outlet pressure (Fig. 1G) to a low input pressure for the RFBI. This intermediate system provided a maximum CO_2_ gas flow of 15 L/min at low pressures (1–4 mmHg) to the RFBI.
- **Primary trocar**: An 11 mm trocar (30103H8, HiCap-Trocar, Karl Storz GmbH & CO. KG, Tuttlingen, Germany) with a high-flow insufflation connector and a mechanical valve served as the main insufflation port. This trocar was selected for its low-resistance insufflation tube connector, as standard Luer lock insufflation connections typically increase flow resistance and thereby limit the ability to maintain stable pressure.
- **Insufflation tubing**: Two filtered insufflation tubes (031200–10, 300 cm, Karl Storz GmbH & CO. KG, Tuttlingen, Germany) were used, one connecting the intermediate CO_2_ delivery system to the gas inlet of the RFBI, the other tube connecting the RFBI gas outlet to the primary trocar in the sterile surgical field (Fig. 1C).

### Sample size calculation

In preclinical experiments, an RFBI prototype demonstrated consistent performance despite physiological and biomechanical variability across 30 porcine procedures [10]. In line with recommendations for pilot studies with continuous variables [11], a sample size of 12 participants was considered sufficient to identify common device-related problems and to evaluate feasibility in a clinical setting. The study was not powered to detect rare adverse device effects. Therefore, this first-in-human trial was designed to enroll 12 participants to evaluate the safety and feasibility of the RFBI during adult laparoscopic surgery.

### Statistical analysis

Acquired data were processed and visualized using MATLAB (version 23.2.0 R2023b, The MathWorks Inc., Natick, Massachusetts, USA) and IBM SPSS Statistics (2021, version 28.0, IBM Corp, Armonk, NY, USA). Descriptive statistics were used for patient characteristics, demographics, and procedure duration.

For assessment of the primary outcomes and intraoperative events affecting pressure stability, all events during surgery were recorded in real time by two independent researchers in accordance with the four-eyes principle and analyzed descriptively. For the secondary outcome of insufflation pressure stability, this was defined as the degree to which the measured pressure remained within a narrow range around the target pressure. Stability was quantified for each target pressure by calculating the median and interquartile range (IQR) of the pressure signal, expressed in mmHg. Stability analyses were stratified by target insufflation pressure, as performance may vary between target pressures as a consequence of radial fan control and rotational speed. Events involving prolonged suctioning were excluded from stability analyses, as maximum CO_2_ supply flow limited performance under these conditions and could bias baseline stability measurements.

## Results

### Patients

Between April and October 2024, thirteen patients were enrolled. One patient was excluded when the surgical approach was changed from laparoscopic to robot-assisted laparoscopic surgery, in which the study trocar could not be used. Twelve patients were therefore included in the analysis. The median age was 70 years (range, 54–79 years), with 83.3% (10/12) being male. Body mass index (BMI) ranged from 18.5 to 34.9 kg/m^2^, with most patients in the 25–30 kg/m^2^ category (41.7%). The American Society of Anesthesiologists (ASA) classification was II in 33.3% of the patients, III in 58.3%, and IV in 8.3%, indicating that most patients had significant systemic comorbidities. 25.0% of the patients had pulmonary conditions, and 33.3% had undergone previous laparoscopic abdominal surgery. Demographic and baseline characteristics are shown in Table 1.

**Table 1:** Patient demographics

	Participants (*N* = 12)
**Age, years (min, max)**	
Median	70.0 (54, 79)
**Gender, n (%)**	
Female	2 (16.7)
Male	10 (83.3)
**BMI (kg/m**_**2**_**), n (%)**	
< 18.5	0 (0)
18.5 – 25	4 (33.3)
25 – 30	5 (41.7)
30 – 35	3 (25.0)
≥ 35	0 (0)
**ASA-classification, n (%)**	
1	0 (0)
2	4 (33.3)
3	7 (58.3)
4	1 (8.3)
5	0 (0)
***Medical history***	
**Previous pregnancy, *n* (%)**	2 (16.7)
1 pregnancies	0 (0)
2 pregnancies	1 (8.3)
3 pregnancies	1 (8.3)
Cesarean section	0 (0)
**Previous laparoscopic surgery°, n (%)**	4 (33.3)
Abdominal wall pathology*, n (%)	3 (25.0)
**Pulmonary conditions^, n (%)**	3 (25.0)

*BMI* Body Mass Index, *ASA* American Society of Anesthesiologists classification system for medical scoring

° specification intraperitoneal surgery: laparoscopic low anterior resection and ileostomy, laparotomy anastomotic leakage, laparoscopic fundoplication hiatus hernia, diagnostic laparoscopy, laparoscopic appendectomy

* specification abdominal wall pathology: correction for hernia umbilicalis, hernia cicatricalis, or hernia inguinalis, and the presence of an ileostoma

^ specification pulmonary conditions: COPD class I

### Surgical procedures

Seven different laparoscopic procedures were performed (Table 2). The majority involved major upper gastrointestinal surgery, including esophageal resection with gastric pull-up reconstruction and subtotal gastrectomy. Other procedures included a combined TAMIS (transanal minimally invasive surgery) and laparoscopic bowel resection, Nissen fundoplication for recurrent hiatal hernia, and abdominoperineal extirpation. The mean total duration per procedure was 299 (range 31–598) minutes, whereas the mean RFBI insufflation time was 129 (range 22–184) minutes, resulting in 1551 min (= 25.85 h) of total insufflation time with the RFBI across the study cohort.

**Table 2:** Surgical procedure details

Procedure	Outcome procedure	Total time surgery (minutes)	Total time abdominal insufflation (minutes)	Pressure stability		
				Target pressure 10 mmHg (median (IQR))	Target pressure 14 mmHg (median (IQR))	
Esophageal resection with gastric pull-up reconstruction, transhiatal laparoscopy with cervical incision	No complications	318	151	10.0 (0.18)		14.0 (0.21)
Combined TAMIS procedure and laparoscopic closed bowel resection of colorectal anastomosis, closing ileostoma and making a terminal colostoma	No complications	158	44	10.0 (0.12)		
Esophageal resection with gastric pull-up reconstruction, thoracic robot-assisted procedure and abdominal laparoscopic surgery	Thorax converted due to pneumomediastinum, abdominal laparoscopic procedure no complications	598	132	10.0 (0.12)		14.0 (0.12)
Nissen fundoplication for recurrent hiatal hernia	No complications	206	184	10.0 (0.12)		14.0 (0.12)
Esophageal resection with gastric pull-up reconstruction, thoracic robot-assisted procedure and abdominal laparoscopic surgery	No complications	416	128	10.0 (0.12)		14.0 (0.16)
Subtotal gastrectomy with Roux-en-Y reconstruction	No complications	272	169			14.0 (0.16)
Abdominoperineal extirpation, robot-assisted procedure	No complications	235	141			14.0 (0.14)
Esophageal resection with gastric pull-up reconstruction, thoracic robot-assisted procedure and abdominal laparoscopic surgery	No complications	493	168			14.0 (0.14)
Esophageal resection with gastric pull-up reconstruction, thoracic robot-assisted procedure and abdominal laparoscopic surgery	By inspection metastases, procedure stopped after taking biopsies	31	22			14.0 (0.14)
Esophageal resection with gastric pull-up reconstruction, transhiatal laparoscopy with cervical incision	No complications	231	131			14.0 (0.14)
Subtotal gastrectomy	Converted due to size tumor	244	142			14.0 (0.14)
Esophageal resection with gastric pull-up reconstruction, thoracic robot-assisted procedure and abdominal laparoscopic surgery	No complications	384	139			14.0 (0.14)

Several procedures required additional non-laparoscopic surgical approaches, e.g., thoracoscopic or transanal surgery, during which the RFBI was not used, as these did not meet the inclusion criteria. Consequently, total operative times exceeded RFBI insufflation times in these cases. Target insufflation pressures of 8, 10, and 14 mmHg were used for different patients and procedures, with intraoperative pressure adjustments made during the procedure to optimize surgical conditions and safety. One example was lowering the insufflation pressure to limit the impact on circulation and ventilation after creating a capnothorax resulting from pleural opening in hiatal dissection during an esophageal resection with gastric pull-up reconstruction. The 11 mm study trocar was inserted below the umbilicus (infraumbilical) using an open introduction technique in 11 patients and a closed introduction in one patient, based on surgeon preference.

### Anesthetic and ventilatory management

All patients received neuromuscular relaxation with rocuronium during insufflation, indicated by a train-of-four (TOF) count of 0/4. Lungs were ventilated in volume-controlled mode for all patients, with the positive end-expiratory pressure (PEEP) set between 5.0 and 10.0 cm H_2_O. Tidal volumes ranged from 4.7 mL/kg (in a patient with moderate pulmonary impairment) to 8.5 mL/kg (410 to 640 mL). Ventilation parameters were adjusted according to the individual patient’s clinical conditions and intraoperative requirements.

### Safety and feasibility

All laparoscopic procedures were completed using the RFBI without conversion to a conventional insufflator and no occurrence of SADEs. One adverse device effect (ADE) was recorded: unintentional loss of surgical field. This was caused by the insertion of a 5 mm instrument into the 11 mm study trocar, creating a large gas leak (> 15 L/min) alongside the instrument, as the used silicone leaflet cap could not provide an airtight seal for the 5 mm instrument. The RFBI could not compensate for this prolonged high leakage rate due to limitations of the CO_2_ gas delivery system, resulting in an unintentional and temporary loss of the surgical work field. There was no harm to the patient or the user of the device. The event was resolved by removing the instrument from the trocar, and the surgeon continued and completed the procedure with the RFBI without further interruption. No other ADEs were observed.

### Insufflation performance

Pressure stability was analyzed at two target pressures: 10 mmHg (264 min of recorded insufflation time) and 14 mmHg (1065 min). Data from insufflation at 8 mmHg were excluded because this pressure setting was applied for only 9 min in total, which was considered insufficient for meaningful analyses. At 10.0 mmHg, the median outlet pressure was 10.0 mmHg (IQR 0.12 mmHg), and at 14.0 mmHg, the median pressure was 14.0 mmHg (IQR 0.15 mmHg).

**Fig. 3:**
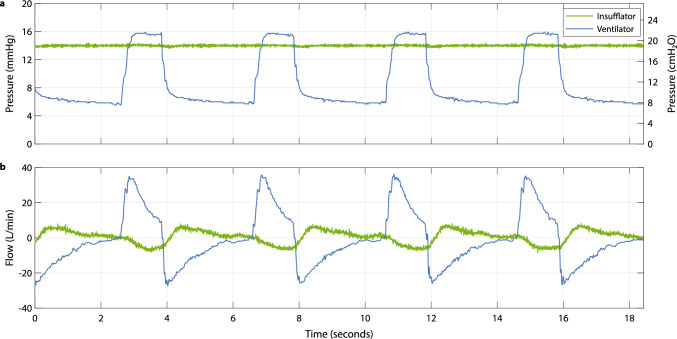
Radial fan-based insufflation technique. A representative recording obtained during insufflation in one of the procedures, illustrating the interaction between mechanical ventilation (purple) and radial fan-based insufflation (green). The upper graph **a** displays mechanical ventilation and insufflation pressure, displayed in mmHg on the left y-axis and in cmH_2_O on the right y-axis (equivalent scaling). The lower graph **b** displays flow of mechanical ventilation and insufflation

Figure 3 illustrates a representative recording of pressure and flow behavior during RFBI insufflation. The example was taken at a target pressure of 14 mmHg under volume-controlled mechanical ventilation with a mechanical ventilation tidal volume of 500 mL, PEEP of 8 cmH_2_O, and a respiratory rate of 12 breaths per minute. During inspiration, the rise in intrathoracic pressure displaced the diaphragm downward toward the abdominal cavity, causing CO_2_ from the abdominal cavity to flow back into the RFBI, as indicated by the negative slope in the insufflation gas flow trace (Fig. 3a). During expiration, pulmonary outflow of air decreased the intrathoracic pressure, moving the diaphragm upward. Gas flowed back from the RFBI reservoir into the abdominal cavity, seen as a positive slope in the insufflation gas flow trace. In this data segment, the volume of gas exchanged between the abdominal cavity and the RFBI with each breath was found to be approximately 103 ml, equivalent to about one-fifth of the mechanical ventilation tidal volume filling the thoracic cavity. Across the recorded ventilation cycles, the insufflation pressure remained within a range of 13.6 to 14.4 mmHg. This demonstrates that ventilation-related pressure fluctuations were limited to less than approximately 0.4 mmHg for most of the respiratory cycle, with no visible lag in the pressure trace despite cyclical bidirectional flow.

### Impact of intraoperative events

Several intraoperative events transiently influenced pressure stability: trocar insertion or repositioning, leaks alongside trocars, (partly) opening valves of disposable trocars, the use of instruments (especially the laparoscopic camera) or materials (drains, suture thread, gauzes) through trocars, the use of suction, entry into an additional cavity (e.g., thorax during a transhiatal approach with pneumothorax), and externally applied pressure on the abdomen by hands or instruments. During these events, the RFBI did not always maintain pressure within the 95% CI range of pressure stability. However, pressure stability recovered rapidly once the event was over, no manual intervention was needed, and the surgeon was not hindered in continuing the procedure, as the duration of the events was generally not more than a few seconds. Importantly, no clinical consequences or complications associated with these fluctuations occurred, and overall device safety and efficacy were preserved throughout all events.

## Discussion

This first-in-human study demonstrates the primary objective, that a radial fan-based CO_2_ insufflator can be safely and effectively used in adult laparoscopic surgery, maintaining a stable capnoperitoneum across a wide range of procedures and surgical conditions. This technology allowed continuous bidirectional gas flow between the abdominal cavity and the device’s integrated gas reservoir, resulting in the device consistently maintaining stable insufflation pressures (median 14.0 mmHg (IQR 0.15); median 10.0 mmHg (IQR 0.12)) despite the challenges of mechanical ventilation and surgical manipulation. No serious adverse device effects occurred, and only one minor event occurred due to incorrect use of the study trocar, without causing harm to the patient or the user.

Previous research has emphasized that continuous insufflation pressure systems outperform conventional insufflation systems in maintaining pressure, especially during suction and leakages [8, 12–14]. The continuous pressure insufflation system EVA15 (Palliare, Galway, Ireland) and valveless trocar system Surgiquest AirSeal Intelligent Flow System (ConMed, Utica, New York, USA) demonstrated improved pressure stability compared to conventional insufflators, with a mean absolute pressure difference of 2.62 mmHg between EVA15 and a conventional insufflator, and 0.27 mmHg between EVA15 and AirSeal [12]. In a randomized trial, valveless insufflation showed greater stability at a target of 15 mmHg (mean 14.0, SD 1.3 mmHg) than conventional insufflation (mean 14.7, SD 1.7 mmHg) [8]. While direct comparability to the current results of the presented study is limited due to different measurement sites (intra-abdominal pressure in previous studies versus device outlet pressure in the current study), the low IQR observed with RFBI suggests effective pressure control at the device level. Further research using intra-abdominal pressure measurements is needed to determine how the RFBI performs in a direct comparison with other insufflation systems under standardized conditions.

During several procedures, the integrated reservoir was observed to reach its maximum capacity of gas filling. This restricted the ability to attenuate intra-abdominal pressure peaks. Although the radial fan continued to regulate pressure by adjusting rotational speed, gas flow restrictions under these circumstances resulted in noticeable pressure fluctuations. Several factors may have contributed to this restriction, including excessive CO_2_ backflow to the device due to high external pressure or a high gas supply from the CO_2_ delivery system. Optimizing the flow regulation mechanisms may further enhance pressure stability during surgery.

The observed ADE highlights both technical and clinical considerations. From a technical perspective, enhancing the CO_2_ gas delivery system to increase its flow capability would enable the device to maintain the target pressure even during high air leakage rates. In addition, providing dedicated silicone leaflet caps for small and large diameter instruments, or a different valve design, could prevent such events. From a clinical perspective, unintentional loss of surgical workspace may also pose a safety risk and prolong the duration of surgery. This underlines the importance of careful instrument–trocar matching and prompt recognition of pressure loss, enabling timely corrective action to minimize disruption and safeguard patient safety.

A strength of this study is the variety of laparoscopic procedures in which the device was tested, overall complex procedures with long durations. In addition, by using the device in a diverse patient population with varying BMI, ASA scores, and medical history in abdominal procedures, we were able to demonstrate its feasibility and adaptability under a broad spectrum of clinical conditions and its suitability for multiple procedures, including a transhiatal approach. When opening the thoracic cavity from the abdominal side, the reservoir provided the ability to insufflate the thoracic cavity within a short time span, preventing collapse of the abdominal cavity through pressure and volume equilibration. Another strength of this study is the high-resolution and simultaneous monitoring of both ventilation and insufflation parameters, enabling a comprehensive assessment of their interaction during the procedure. A limitation is that insufflation pressure was measured at the device outlet, without accounting for potential pressure differences caused by the resistance of the insufflation circuit. This could result in pressure discrepancies between the measured and actual pressure within the abdominal cavity, which we calculated to be approximately 2 mmHg based on an in vitro test. The majority of insufflators measure pressure within the device and are likely to suggest a more stable pressure than actually exists within the abdomen. Direct measurement of intra-abdominal pressure could provide additional value for a more thorough analysis of stability. This study included a limited number of patients to assess the safety and feasibility of the device, rather than the clinical effectiveness. These data can be used to design a larger comparative study that is more specifically powered for clinical effectiveness. Further research will focus on directly comparing RFBI performance with conventional insufflation measuring the intra-abdominal pressure, as well as on the potential clinical impact of stable pressure and the attenuation of pressure peaks caused by mechanical ventilation. Stabilization may contribute to improved surgical field stability for the surgeon and to increase pulmonary compliance due to reduced intra-abdominal pressure and decreased resistance to mechanical ventilation. The surgical workspace can be comparatively investigated using numeric rating scales [15]. Patient outcomes can be investigated by focusing on perioperative variables (e.g., duration of surgery and ventilation pressure peaks) and postoperative variables (e.g., recovery time, postoperative pain, and complication rates). In addition, we aim to evaluate the device in specific patient subgroups, such as pediatric and obese patients, as these populations may derive particular benefit from this novel insufflation technique.

In conclusion, this first-in-human study demonstrates that our radial fan-based CO_2_ insufflator is safe and feasible for use during intraperitoneal laparoscopic surgery in adults. The technique provided stable insufflation pressures and adequate compensation for the interaction with ventilation pressures. This clinical trial serves as a prelude to future assessments of its potential impact on clinical outcomes.
